# mGluR5 Ablation in Cortical Glutamatergic Neurons Increases Novelty-Induced Locomotion

**DOI:** 10.1371/journal.pone.0070415

**Published:** 2013-08-05

**Authors:** Chris P. Jew, Chia-Shan Wu, Hao Sun, Jie Zhu, Jui-Yen Huang, Dinghui Yu, Nicholas J. Justice, Hui-Chen Lu

**Affiliations:** 1 The Cain Foundation Laboratories, Baylor College of Medicine, Houston, Texas, United States of America; 2 Jan and Dan Duncan Neurological Research Institute at Texas Children’s Hospital, Baylor College of Medicine, Houston, Texas, United States of America; 3 Department of Pediatrics, Baylor College of Medicine, Houston, Texas, United States of America; 4 Huffington Center on Aging, Baylor College of Medicine, Houston, Texas, United States of America; 5 Program in Developmental Biology, Baylor College of Medicine, Houston, Texas, United States of America; 6 Department of Neuroscience, Baylor College of Medicine, Houston, Texas, United States of America; Max Planck Institute of Psychiatry, Germany

## Abstract

The group I metabotropic glutamate receptor 5 (mGluR5) has been implicated in the pathology of various neurological disorders including schizophrenia, ADHD, and autism. mGluR5-dependent synaptic plasticity has been described at a variety of neural connections and its signaling has been implicated in several behaviors. These behaviors include locomotor reactivity to novel environment, sensorimotor gating, anxiety, and cognition. mGluR5 is expressed in glutamatergic neurons, inhibitory neurons, and glia in various brain regions. In this study, we show that deleting mGluR5 expression only in principal cortical neurons leads to defective cannabinoid receptor 1 (CB1R) dependent synaptic plasticity in the prefrontal cortex. These cortical glutamatergic mGluR5 knockout mice exhibit increased novelty-induced locomotion, and their locomotion can be further enhanced by treatment with the psychostimulant methylphenidate. Despite a modest reduction in repetitive behaviors, cortical glutamatergic mGluR5 knockout mice are normal in sensorimotor gating, anxiety, motor balance/learning and fear conditioning behaviors. These results show that mGluR5 signaling in cortical glutamatergic neurons is required for precisely modulating locomotor reactivity to a novel environment but not for sensorimotor gating, anxiety, motor coordination, several forms of learning or social interactions.

## Introduction

The group I metabotropic glutamate receptor 5 (mGluR5) is a G protein-coupled receptor that primarily signals through Gα_q/11_ and modulates various kinases, ion channels, and intracellular calcium stores through DAG and IP3 signaling (reviewed in [1], [2]). At many mature synapses, mGluR5 activity in glutamatergic synapses triggers the synthesis of 2-arachidonoyl glycerol (2-AG) [3], [4], [5], [6], one of the major endocannabinoids (eCBs), to modulate presynaptic function through cannabinoid receptor 1 (CB1R) [3], [4], [5], [6], [7], [8], [9]. mGluR5 dependent synaptic plasticity has been found in a diverse range of synaptic connections [9], [10], [11], [12] and mGluR5 signaling has been implicated in the pathology of several significant neurological disorders, including Fragile X syndrome (FXS) [13], [14], [15], [16], [17], [18], attention-deficit/hyperactivity disorder (ADHD) [19], [20], autism [21], [22], schizophrenia [2], [19], [23], and epilepsy [24].

mGluR5 has been proposed to play a role in several behaviors including in locomotor reactivity to novel environment, sensorimotor gating, anxiety, and cognition [25], [26], [27], [28], [29], [30], [31], [32]. Global mGluR5 knockout (KO) mice are hyperactive in novel environments [33], [34], [35] and defective in the prepulse inhibition of the acoustic startle reflex [30], [31], [32]. Anxiety-like behaviors and learning are also altered in global mGluR5 KO mice [35], [36]. Interestingly, acute blockade of mGluR5 signaling using the noncompetitive mGluR5 antagonist 2-methyl-6-(phenylethynyl)-pyridine (MPEP) induces hyperactivity in wildtype mice [37] and produces anxiolytic as well as anti-depressive effects [25], [29], [38], [39].

mGluR5 expression in adult mouse brain is very high in the olfactory bulb, cortex, striatum, and hippocampus ([40] and http://mouse.brain-map.org), where it is present in glutamatergic neurons, inhibitory neurons, and glia [40], [41], [42], [43], [44], [45], [46]. In previous studies we demonstrated that mGluR5 signaling in cortical glutamatergic neurons is required for regulating the excitability of cortical neurons in primary somatosensory (S1) cortex [47]. In this study, we first examined the induction of long-term depression within the prefrontal cortex of adolescent Cx-mGlu5 KO mice to determine the contribution of cortical glutamatergic mGluR5 in synaptic plasticity. Next, Cx-mGlu5 KO mice and their littermate controls were subjected to a panel of behavioral assays to explore the contribution of mGluR5 signaling in cortical glutamatergic neurons to novelty-induced locomotion, anxiety, sensorimotor gating, motor coordination and learning, social interaction, as well as perseverative behaviors. The results help to define the roles of mGluR5 signaling in specific neuronal populations and circuits.

## Materials and Methods

### Animal and Genotyping

mGluR5 floxed (mGluR5^f/f^) mice in a mixed 129 SVJ and C57BL/6 background (129/C57) were generated as described in Xu et al., (2009) [48]. NEX-Cre mice in a mixed 129 SVJ and C57BL/6 background were generated by knocking the Cre gene into the NEX locus [49]. NEX-Cre/+;mGluR5^f/f^ males were mated with mGluR5^f/f^ females to produce NEX-Cre/+;mGluR5^f/f^ (Cx-mGlu5 KO) and +/+;mGluR5^f/f^; (control) mice. To minimize the effects of mixed genetic background, littermate controls were used for all experiments. Furthermore, 129 SVJ inbred mice are known for their high innate anxiety levels [50], [51]. Therefore, Cx-mGlu5 KO mice in the low anxiety C57BL/6 background (C57) were generated for use in selected experiments as follows: Mice from the original mGluR5^f/f^ were back-crossed with C57BL/6 mice for eight generations. NEX-Cre/+ mice were backcrossed into C57BL/6 background for five generations. The congenic strains produced after back-crossing were crossed to generate NEX-Cre/+;mGluR5^f/f^ and +/+; mGluR5^f/f^ mice. Genotyping was conducted with the procedure previously described [47]. Mouse colonies were maintained in a pathogen-free environment with a 14–10 hour light:dark cycle (lights on at 6∶00 AM) with access to food and water *ad libitum*. All experiments and data analysis were done blind to genotype information. Animals were treated in compliance with the U.S. Department of Health and Human Services and Baylor College of Medicine guidelines. The Baylor institutional Animal Care and Use Committee (IACUC) approved this study.

### Western Blot Analysis

Cortices were collected from postnatal day 4 (P4), P14, and 6-month-old mice, while brain stem, spinal cord and striatum were harvested from 6-month-old mice. These tissues were homogenized with modified radio immunoprecipitation assay (RIPA) buffer (50 mM Tris-base pH 7.4, 50 mM NaCl, 1% Triton X-100, 0.1% SDS, 1 mM EDTA, 1% Na-deoxycholate) with protease and phosphatase inhibitor mixture tablets (Roche Applied Science, Indianapolis, IN, USA). Protein concentrations were determined using the Bradford assay (Bio-Rad, Hercules, CA, USA), and 20–25 µg of total protein was electrophoretically separated on 4–15% gradient SDS-PAGE gels and transferred onto nitrocellulose membranes (Criterion system from Bio-Rad) or polyvinylidene difluoride (PVDF) membrane (Bio-Rad). Membranes were blocked with 3% bovine serum albumin (BSA) in Tris-buffered saline (TBS, 20 mM Tris-HCl, pH7.5, 150 mM NaCl) with 0.2% Tween 20 (TBS-T) for 1 hour at room temperature, then probed with rabbit anti-β-actin (Sigma, St. Louis, MO, USA; 1∶10000), rabbit anti-glyceraldehyde 3-phosphate dehydrogenase (GAPDH) (Cell Signaling Technology, Danvers, MA, USA; 1∶5000), rabbit anti-mGluR1 antibody (Millipore, Billerica, MA, USA; 1∶1000), or rabbit anti-mGluR5 antibodies (Millipore; 1∶2000) in blocking buffer for 16–18 hours at 4°C. Membranes were then washed with TBS-T for 3 times with 5 minutes each time, and incubated with horseradish peroxidase-conjugated anti-rabbit secondary antibody (Jackson Immunoresearch Laboratories, West Grove, PA, USA; 1∶10000) in TBS-T for 1–2 hours at room temperature, followed by washings in TBS-T for 4 times with 10 minutes each time. Immunoreactivity was visualized with WEST PICO chemiluminescence reagent or Super Signal West Dura Chemiluminescent substrate (Thermo Fisher Scientific In., IL, USA) according to manufacturer’s instruction, and chemiluminescence signals were detected and digitized with ImageQuant LAS 4000 (GE Healthcare, Little Chalfont, UK). Densitometric quantification was performed using ImageJ software (NIH, Bethesda, MA, USA). The expression levels of each protein of interest were first normalized to that of β-actin or GAPDH, and then normalized to the mean value of the corresponding littermate control for each brain region examined at each age.

### Multiple Immunofluorescent Staining

Immunofluorescence labeling was performed on four wild-type and four Cx-mGlu5 KO (129/C57 mixed) mice (2 months of age). Under deep anesthesia with rodent comboanesthetic III (2.5 µl/g i.p.), mice were perfused intracardially with 4% ice cold paraformaldehyde in PBS, pH 7.4. The brains were removed and fixed in 4% paraformaldehyde overnight at 4°C. Fixed brains were sectioned into 100 µm thick sections in the coronal plane. Sections were washed with PBS-T (PBS with 0.01% Triton X-100) and permeabilized with 0.3% Triton X-100 in PBS at room temperature for 20 minutes. Sections were then washed with PBST, blocked for 1 hour with 3% normal goat serum in PBS with 0.3% Triton X-100 at room temperature, and then incubated with a mixture of rabbit anti-mGluR5 (Millipore; 1∶2000) and guinea pig anti-CB1 (Cannabinoid receptor type 1) (gift from Dr. Ken Mackie; 1∶1000) in PBS-T with 2 mg/ml BSA and 1% normal goat serum at 4°C overnight. The next day, sections were washed with PBS-T, and incubated with the mixture of secondary antibodies: goat anti-guinea pig IgG-Alexa 488 (Invitrogen, Grand Island, NY, USA; 1∶500), goat anti-rabbit IgG-Alexa 594 (Invitrogen; 1∶500), in PBST at room temperature for 2 hours. Following this incubation, sections were washed with PBST twice for 5 minutes each. After DAPI staining (Invitrogen; 1∶10,000 ng/ml in PBS for 10 minutes) to identify nuclei, sections were washed three times for 10 minutes each and mounted onto Superfrost Plus slides (Fisher Scientific, Waltham, MA, USA), cover-slipped with Vectashield (Vector, Burlingame, CA, USA) and sealed with nail polish for confocal or fluorescent imaging.

### Imaging

Fluorescent images were taken with a Zeiss AxioImager M1 system (Oberkochen, Germany) with 5×/0.16 Zeiss objectives, using AxioVision software. Confocal images were obtained using a Zeiss 710 system with 10×/0.3, 20×/0.5, 40×/1.3 (oil) objective lens. Alexa 488, Alexa 594, DAPI fluorophores were excited with lasers of appropriate excitation wavelength sequentially (488 nm, 561 nm, or 405 nm) and scanned with emission filters selected to optimally separate fluorescence. All corresponding images were acquired from the same setting and processed as a whole in Adobe Photoshop CS2 (Adobe Systems, San Jose, CA, USA) for brightness/contrast, orientations, and background corrections to better illustrate the staining patterns.

### *In vitro* Electrophysiology and Data Analysis

Brain slices containing the medial PFC were obtained from 4–6 week old mice (in 129/C57 mixed background). Coronal brain slices (400 µm) at 3.0–3.2 rostral to bregma were cut in ice-cold modified artificial cerebrospinal fluid (ACSF) containing the following (in mM): 94 sucrose, 30 NaCl, 4.5 KCl, 1 MgCl_2_, 26 NaHCO_3_, 1.2 NaH_2_PO_4_, and 10 D-glucose and adjusted to pH 7.4 by bubbling with 95% O_2_/5% CO_2_. Slices were recovered at 32°C for an hour in a holding chamber and recorded at 32–34°C in oxygenated ACSF (composition in mM: 126 NaCl, 2.5 KCl, 1.2 NaH2PO_4_, 2.4 MgCl_2_, 1.2 CaCl_2_, 18 NaHCO_3_, and 11 glucose). The superfusion medium contained GABAzine (SR-95531; 10 µM; Tocris, Minneapolis, MN, USA) to block GABA_A_ receptors. To evoke the field excitatory postsynaptic potential (fEPSP), stimuli (0.1 msec) were delivered through a glass electrode filled with ACSF and placed in layer 2/3. A recording pipette was filled with ACSF and placed in layer 5/6. After baseline recordings (20 minutes, 0.05 Hz), an induction stimulation protocol (10 minutes at 10 Hz) was applied to induce LTD and fEPSPs recorded for a further 40 minutes. The magnitude of LTD was measured as the percentage of the average fEPSP amplitudes 21–40 minutes after stimulation) to the average fEPSP amplitude 20 minutes before stimulation (mean ± SEM %). The glutamatergic nature of the extracellular fEPSP was confirmed at the end of the experiments through the application of the non-NMDA ionotropic glutamate receptor antagonist CNQX (20 µM; Tocris Bioscience, Bristol, UK).

### Pharmacology

2-methyl-6-(phenylethynyl)pyridine (MPEP) hydrochloride was obtained from Tocris and dissolved in 0.9% saline at 4 mg/ml prior to testing. MPEP is a selective noncompetitive antagonist of mGluR5. threo-Methyl α-phenyl-α-(2-piperidyl)acetate (methylphenidate) hydrochloride was obtained from Sigma and dissolved in 0.9% saline at 1 mg/ml prior to testing. Methylphenidate, commonly known as Ritalin, is an amphetamine derivative that acts primarily as a norepinephrine-dopamine reuptake inhibitor. MPEP was injected at a dose of 40 mg/kg i.p., while methylphenidate was injected at a dose of 8 mg/kg i.p. To measure motor effects induced by drugs, separate cohorts of mice were randomly assigned to treatment groups. Each mouse was subjected to no more than 4 treatments, each separated ≥48 hours. MPEP is active for <2 hours for both mouse marble burying behavior and audiogenic seizure assay [52], [53], while Ritalin’s effects on mouse locomotor activity lasts for less than 12 hours, based on our homecage activity studies (data not shown).

### Corticosteroid Levels Measurement

A separate cohort of mice was used to examine circulating corticosteroid levels at 4–5 months of age. To measure circulating corticosteroid levels at rest, animals were placed in a quiet room in their home cages for 2 hours without disturbance. After 2 hours, blood was collected by retro-orbital eye bleed. All bleeds were performed at the same time of day to sample corticosteroids at the equivalent point in the diurnal rhythm of corticosteroid release. Collected blood was placed in a tube containing EDTA, mixed, and centrifuged for 5 minutes. Plasma was removed and kept frozen until analysis. Corticosteroids in plasma samples were measured using a Milliplex Rat Stress Hormone panel immunoassay (RSH69K-01 from Millipore), and read on a Bioplex 200 luminex instrument (Bio-Rad, Hercules, CA, USA).

### Behavioral Testing

Male and female mice at 2–4 months of age were subjected to selected tests from the test battery originally described by Crawley and Paylor [54]. Assays were performed in the order of least stressful to most stressful to the animals. Mice were rested in their home cages for at least one week before being tested in the next assay [55], [56]. For pharmacological treatment for the open field assay, mice were rested for at least two days. One batch of Cx-mGluR5 KO mice in a 129/C57 mixed background was subjected to open field assays, prepulse inhibition, and rotarod assays (in the order listed). One batch of Cx-mGluR5 KO mice in a 129/C57 mixed background was subjected to prepulse inhibition assays and open field with pharmacological treatment. One batch of Cx-mGluR5 KO mice in a 129/C57 mixed background was subjected to open field assay with pharmacological treatment and homecage activity assay. To assess anxiety and behaviors where high innate anxiety may be a confounder, the following behavior assays were performed on Cx-mGluR5 KO and littermate WT mice in C57 background, an inbred mouse strain with lower anxiety levels than 129 SVJ strain [50], [51]. One batch of Cx-mGluR5 KO mice in a C57 background was subjected to the elevated plus maze, open-field, prepulse inhibition, 3-chamber interaction, marble burying, and conditioned fear assays. One batch of Cx-mGluR5 KO mice in a C57 background was subjected to the elevated plus maze, open-field, light-dark exploration, 3-chamber interaction, and marble burying assays. Information on animal numbers used for each behavioral paradigm can be found in the following methods section, as well as in the Tables. Each mouse was subjected to each behavior paradigm once, i.e. naïve to each test, except for the pharmacology experiments where injection and open field assay was repeated for each mouse (less than 4 times total).

Prior to any behavioral testing, mice were allowed to acclimatize to the testing room for at least 30 minutes. Testing was performed under standard room conditions, approximately 750–800 lux of illumination [57], [58]. 55 dB of white noises were used in the assays testing for anxiety (elevated plus maze, light/dark exploration, open field) and in those where anxiety could be a confounder (marble burying, 3-chamber interaction, PPI (70 dB white noise was used), to reduce audio interference from the experimenter or environment [58]. Behavioral testing was performed between 10∶00 AM and 4∶00 PM (mid phase of the light cycle). Experimenters were blind to the genotypes of the mice.

### Elevated Plus-maze

The elevated plus-maze was made of four perpendicular runways (7×25 cm) elevated 40 cm off the ground. Two arms were enclosed by 15 cm white walls and two arms were open, except for a low 5 mm rim. The test animals were placed in the center of the elevated maze facing one of the two open arms, and left to explore for 10 minutes [59]. The number of entries, distance traveled and time spent in the open and closed arms were recorded using the ANY-maze tracking system (Stoelting Co., Wood Dale, IL, USA). The number of rearings and groomings in the open and closed arms, and the number of head dips in the open arms were also scored by the experimenter. Cx-mGluR5 mice in C57BL6 background were used for this assay (WT male, n = 10; KO male, n = 9; WT female, n = 4; KO female, n = 4).

### Open Field Exploration

Each mouse was placed into the center of a clear Plexiglas chamber (40 cm×40 cm×30 cm) with photo beams to record its horizontal and vertical movements in an open-field assay (OFA). Activity was recorded using a computer-operated VersaMax Animal Activity Monitor System (Accuscan Instruments, Columbus, OH). Testing was performed in the presence of overhead bright lights (∼750 lux of illumination) and white noise (55 db) [54], [57], [59]. These lighting and white noise conditions allowed us to examine anxiety-related responses in addition to locomotion. Before all open field experiments, mice were individually weighed, tails marked, and introduced into the testing room to acclimate for at least 30 minutes before testing. Data was collected in 10-minute interval bins and the following measures were analyzed: total distance traveled, time spent traveling, horizontal traveling speed, vertical activity, center zone distance traveled, center zone time, center zone entries, and center distance ratio. The center zone is defined as an unmarked square area (22.5 cm×25.5 cm) lying in the middle of the arena. Horizontal traveling speed was calculated by dividing total distance traveled by time spent traveling for individual time bins. The ratio of center distance to total distance ratio was used as a measure of anxiety-related response to the brightly lit open area in the center of open field arena [54]. Data for all experiments presented in a line graph represents aggregate scores over each 10-minute bin. For WT and KO comparisons, pooled data represent the combined scores over the entire 30-minute period. Both Cx-mGluR5 mice in 129/C57 background (WT male, n = 15; KO male, n = 15; WT female, n = 16; KO female, n = 13) and Cx-mGluR5 mice in C57BL6 background were tested (WT male, n = 16; KO male, n = 17; WT female, n = 6; KO female, n = 7).

For MPEP and methylphenidate experiments, only male mice were used and each mouse was tested with drug and corresponding vehicle in separate OFA assays. They were first placed inside a Plexiglas chamber and allowed to habituate and to be recorded for 60 minutes. After the habituation period, the mice were returned back into their original cages with access to food and water while their Plexiglas chambers were cleaned. Each mouse was then given a single i.p. injection and immediately placed back into its original Plexiglas chamber for a 60-minute recording period. The locomotion changes in response to MPEP or methylphenidate treatment were calculated to compare the response to drug relative to their vehicle controls in the open field assay. Increased locomotion was triggered by i.p. vehicle injections in both WT and Cx-mGluR5 KO mice. This enhanced activity by vehicle injections lasted 15–30 minutes. Thus, we focused our data analysis to examine changes in locomotion due to drug treatment by analyzing OFA data 31–40 minutes after i.p. injections. A drug response index was defined as the ratio of the total distance traveled during the 31–40 minutes after drug injection to the average total distance traveled during the 31–40 minutes after vehicle injection. The average of the vehicle response was calculated by averaging the total distance during 31–40 minutes after vehicle administration from individual animals of the same genotype. A few animals exhibit zero movement 31–40 minutes after vehicle injection. Thus, the average value was used to calculate the response index instead of using paired individual values. Response indexes were compared using unpaired student’s t-test. Cx-mGluR5 mice in 129/C57 background were examined for MPEP/vehicle (20 WT males and 15 KO males) and methylphenidate/vehicle (9 WT males and 12 KO males).

### Acoustic Startle and Prepulse Inhibition (PPI) of Acoustic Startle

Prepulse inhibition of the acoustic startle response was determined as described [57], [60]. Briefly, acoustic startle responses were measured using the SR-Lab startle response system (San Diego Instruments, San Diego, CA, USA). Each mouse was placed in a Plexiglas cylinder within a sound-attenuating chamber and habituated to a 70-dB background white noise for 5 minutes prior to beginning the test session. Each test session consisted of six blocks, with each block containing eight pseudo-randomized trial types. These include: no stimulus (to measure baseline movement), startle stimulus only (120-dB, 40 msec), and three prepulse stimuli (74, 78, 82-dB; 20 msec) presented either alone or 100 msec before the startle stimulus [58]. The inter-trial intervals ranged from 10 to 20 seconds. Startle responses, detected as force changes within the Plexiglas cylinder, were recorded every 1 msec during a 65 msec period that followed the onset of either the prepulse during prepulse-alone trials or the startle stimulus. The maximum startle amplitude was used as the dependent variable. Percent PPI of the startle response was calculated for each prepulse as 100 - [(startle response to trials with prepulse and startle stimulus trials/startle response to trials with startle stimulus alone) ×100]. Cx-mGluR5 mice in 129/C57BL6 background (WT male, n = 25; KO male, n = 20; WT female, n = 14; KO female, n = 14) and Cx-mGluR5 mice in C57BL6 background (WT male, n = 8; KO male, n = 8; WT female, n = 9; KO female, n = 9) were tested.

### Homecage

Mice were individually placed in standard polypropylene cages (16.5×27 cm) similar in size to their holding cages, and their horizontal locomotor activities were assessed by an automated recording system (San Diego Instruments, San Diego, CA) as described [61]. These cages are very similar to their regular housing cages. The cages were placed into frames equipped with five infrared photocell beams (5 cm apart). Locomotion was measured as the number of sequential breaks in two adjacent beams. The locomotion of test animals was monitored for three days and data acquired from the first few hours were discarded. Cx-mGluR5 mice in 129/C57BL6 background were tested (WT male, n = 14; KO male, n = 14).

### Rotarod Test

Motor coordination and skill learning were tested using an accelerating rotarod (UGO Basile, Varese, Italy) [57]. Mice were placed on a rotating drum (3 cm in diameter), which accelerated from 4 to 40 rpm over a 5-minute period. The time spent walking on top of the rod until the mouse either fell off the rod, or slipped and held onto the rod to ride completely around was recorded. Mice were given four trials per day on 2 consecutive days with a maximum time of 300 seconds (5 minutes) per trial and a 60 minute inter-trial rest interval. The averaged data for each day were analyzed [59]. Cx-mGluR5 mice in 129/C57BL6 background were tested (WT male, n = 11; KO male, n = 10).

#### Parallel rod footslip test

The test apparatus (Stoelting) was enclosed in a clear Plexiglas chamber (20 cm×20 cm×28 cm); 1.6 mm diameter rods were spaced 6 mm apart and elevated 1 cm above a metal plate [59]. In this assay, each mouse was required to walk and balance on thin (1.6 mm) parallel rods spaced 6 mm apart [58]. Ataxia and locomotor activity were recorded simultaneously for 10 minutes. Footslips were detected when a paw touched a metal plate below the parallel rod floor, completing a circuit that was scored by the system. Locomotor activity was measured by the ANY-maze tracking system. The number of errors (footslips) per distance traveled (m) was calculated. Cx-mGluR5 mice in C57BL6 background were tested (WT male, n = 10; KO male, n = 8).

#### Pavlovian conditioned fear

Freezing behavior in a conditioned fear paradigm was measured as described previously [57], [62]. The test chamber (26 cm×22 cm×18 cm high) had clear Plexiglas sides and a grid floor bottom that was used to deliver a mild foot shock. The chamber was placed inside a sound-attenuated chamber (Med Associates, St. Albans, VT, USA) that had a window through which mice could be observed without disturbance. On the training day, mice were placed into the test chamber (house lights ON) and allowed to explore for 2 minutes. The conditioned stimulus (CS, a 80 dB white noise) was presented for 30 seconds and followed immediately by a mild foot shock (2 seconds, 0.7 mA) that served as the unconditioned stimulus (US). Two minutes later, a second CS-US pairing was presented. The FreezeFrame2 monitor system (Actimetrics, Wilmette, IL, USA) was used to control the timing of the CS and US presentations and to measure freezing behavior. In the present study, all of the mice responded to the foot shock.

Mice were tested for contextual and cued fear conditioning 24 hours after conditioning. For the context test, mice were placed back into the original test chamber for 5 minutes and freezing behavior was recorded. One to two hours later, mice were tested for responses to the auditory CS in a new environment. For the CS test, white Plexiglas inserts were placed on the sides and floor of the chamber to alter the shape, texture and color of the chamber. Vanilla extract was placed in the chamber behind the insert to alter the odor. Transfer cages were altered (no bedding) and red house lights replaced the normal white house lights. Mice were placed into this new chamber and freezing was recorded for 3 minutes during this ‘pre-CS’ phase. The auditory CS was then presented for another 3 minutes and freezing was recorded. Data for the cued test were calculated as total percent freezing time. Data for the CS test were calculated as the percent freezing during the CS minus percent freezing in the pre-CS phase. Cx-mGluR5 mice in C57BL6 background were tested (WT male, n = 9; KO male, n = 9; WT female, n = 8; KO female, n = 8).

### Light-dark Exploration Test

Anxiety-like behavior was tested in the light-dark exploration test using a Plexiglas chamber (44×21×21cm) divided unequally into two chambers by a black partition containing a small opening [50], [57]. The larger chamber is twice the size of the smaller chamber, has clear walls and an open top and is brightly illuminated. The small chamber is enclosed on all sides by black walls except for the small opening between the chambers. White noise was present in the room at 55dB in the test chamber. Each mouse was placed into the illuminated side and allowed to explore freely for 10 minutes. The number and latency of entries and time spent in each compartment were scored and analyzed. Cx-mGluR5 mice in C57BL6 background were tested (WT male, n = 10; KO male, n = 14; WT female, n = 10; KO female, n = 11).

### Marble Burying

Before test, clean cages (27×16.5×12.5 cm) were filled with approximately 5 cm deep corncob bedding, then laced with 20 black glass marbles (15 mm diameter) on the surface, evenly spaced in a 4×5 arrangement. Testing consisted of a 30 minutes exploration period [63]. The number of marbles buried (>50% marble covered by bedding material) was counted. White noise (55 dB) was present during testing. Cx-mGluR5 mice in a C57BL6 background were tested (WT male, n = 11; KO male, n = 14; WT female, n = 11; KO female, n = 12).

### 3-Chamber Interaction

The test was performed as previously described [58], [64] with a slight modification of the habituation phase. The 3-chamber apparatus is a clear Plexiglas box (24.75×16.75×8.75 in) with removable partitions separating the box into left, center, and right chambers. One cylindrical wire cage (3 in diameter×4 in height) was placed open end down into the left chamber and one in the right chamber. The chambers of the apparatus and wire cages were cleaned with 30% isopropanol and dried with paper towels between each trial. The habituation and sociability phases were performed as follows:

Mice were subjected to two ten-minute phases within the 3-chamber maze; the first was a habituation phase and the second phase was the social phase. During the habituation phase, the test mouse was placed in the middle chamber and allowed to explore for 10 minutes, with the doorways into the two side chambers open. Each of the two side chambers contained an empty wire cage. The total time the test mouse spent in each side chamber during the 10-minute habituation period was recorded, and the preference ratio calculated. To confirm the absence of a side preference bias for either of the two side chambers of the test box, measures were taken of the total time the test mouse spent in each side during the 10-minute habituation period. After the habituation period, the subject mouse was enclosed in the center compartment of the social test box and the doors to the left and right side chamber were closed. For the social phase, a novel partner mouse was placed into one of the wire cages and an inanimate object of similar size to a mouse was placed in the other empty wire cage, serving as a control with no social valence. The subject mouse was allowed to explore the entire social test apparatus for 10 minutes. Measurements were recorded for the total amount of time spent in each chamber, the total amount of time spent exhibiting directed interest such as sniffing, pawing, or rearing at each wire cage, and basic locomotor activity. Prior to the test, the age- and sex-matched C57BL/6 novel partner mice (adult males) were housed in cages separate and distinct from the cages housing the test mice, to avoid visual, auditory and olfactory contact. Previous to the start of the social testing, the novel partner mice were habituated to the wire cages in the social apparatus for 1 hour per day for two days.

Distance traveled, time spent traveling, and transitions between chambers were recorded. The chamber preference ratio was calculated as the ratio of time the mouse spends in the chamber of the partner mouse vs. the novel object chamber. A second corroborative measure, the sniffing preference ratio was calculated as the ratio of time spent sniffing the cup containing the partner mouse compared with the time spent sniffing the novel object cup. Cx-mGluR5 mice in C57BL6 background were tested (WT male, n = 21; KO male, n = 20).

### Statistical Analyses

Statistical analysis was conducted using SPSS (SPSS, Chicago, IL, USA), Prism 3.0 (Graph-Pad Software) or SigmaPlot (Systat Software Inc., San Jose, CA). All data were first analyzed by two-way ANOVA (gender × genotype) or (genotype × drug), three-way ANOVA (gender × genotype × time or gender × genotype × prepulse sound level for PPI) with repeated measures. In cases where only male mice were tested, student’s t-test (genotype) and two-way ANOVA (genotype × day for Rotarod) with repeated measures were used. When there was no genotype × gender interaction, gender data were combined for statistical comparisons. When there was significant genotype × time interaction effect but no significant gender × genotype effects, gender data were pooled and data analyzed with Student’s t-test for each time point. All data are presented as mean ± SEM. The level of significance was set at p<0.05. Student’s t-tests were also used to analyze the data from Western analysis and electrophysiology recording data.

## Results

### Cortical-specific mGluR5 Deletion in Conditional Knock-out Mice

**Figure 1:**
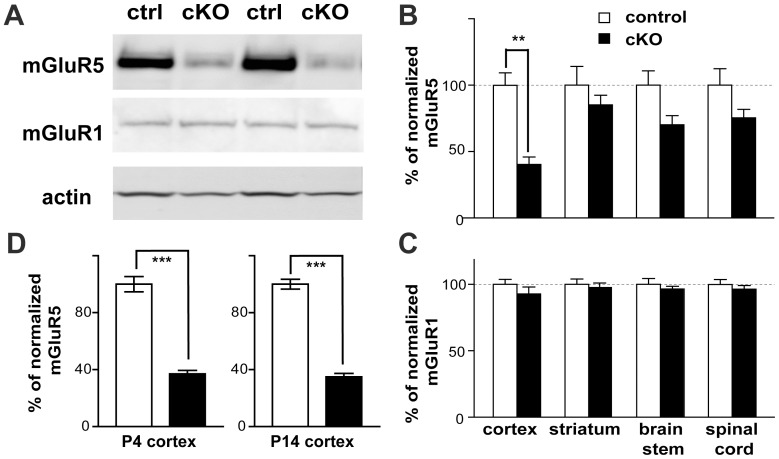
Reduced mGluR5 expression in the cortex of Cx-mGluR5 KO mice. (**A**) Examples of the Western blots used to quantify the abundance of mGluR5, mGluR1 and β-actin in cortex isolated from 6-month old Cx-mGluR5 KO mice and their littermate controls. (**B,C**) Summaries for the levels of mGluR5/β-actin (**B**) and mGluR1/β-actin (**C**). (**D**) Summaries show reduced mGluR5 levels in the developing cortex of Cx-mGluR5 KO mice. Data are presented as mean ± SEM as % of normalized mGluR5 or mGluR1 in control mice for each specific brain region (**, p<0.01, ***, p<0.0001, Student’s t-test).

The cortical glutamatergic-specific mGluR5 KO (Cx-mGlu5 KO) mice were generated by crossing mGluR5 floxed (mGluR5^f/f^) mice [48] with NEX-Cre mice [49] as described [47]. To quantify mGluR5 abundance in each brain area, Western blot analysis was conducted with the cortex, striatum, brain stem and spinal cord prepared from 6-month-old Cx-mGlu5 KO (n = 4) and their littermate control (n = 4) mice (**Fig. 1A–C**). The protein level of mGluR5 in the cortex of adult Cx-mGlu5 KO mice (**Fig. 1A,B**) was 40.29±5.62% of their littermate controls (100±9.33; p<0.01 between WT and KO mice). However, no significant changes in mGluR5 expression were detected in the striatum, brain stem, or spinal cord of Cx-mGlu5 KO cortex (striatum: WT = 100±14.22%, KO = 85.18±7.25%; brain stem: WT = 100±10.82%, KO = 70.24±6.82%; spinal cord: WT = 100±12.34%, KO = 75.42±6.38%). No alteration in the expression of mGluR1, another group I mGluR, was detected in adult Cx-mGlu5 KO mice (**Fig. 1**C; cortex: WT = 100.0±3.87%, KO = 92.84±5.25%; striatum: WT = 100±4.09%, KO = 97.72±3.36%; brain stem: WT = 100±4.49%, 96.62±1.94%; spinal cord: WT = 100±3.72%, KO = 96.43±2.69%). The reduction of mGluR5 expression in the cortex was already evident at P4 and P14 (**Fig. 1D**; P4: WT = 100±5.34%, KO = 37.08±2.37%; P14: WT = 100±3.41%, KO = 34.94±2.40%; n = 5 for each group; p<0.0001 between WT and KO for both P4 and P14).

### Synaptic Plasticity in the Prefrontal Cortex of Cx-mGluR5 KO Mice is Impaired

**Figure 2:**
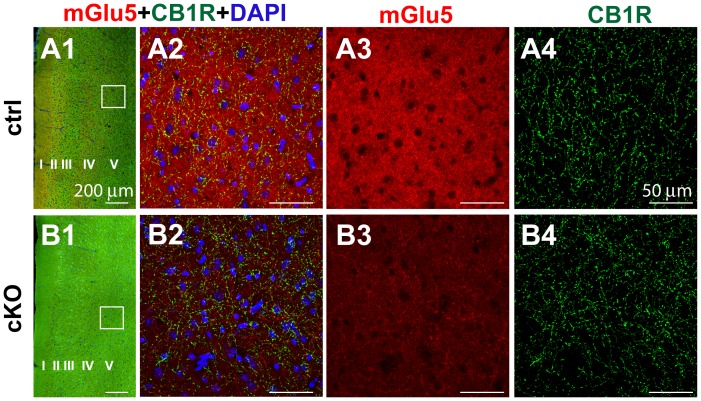
Reduced mGluR5 expression in the layer V of mPFC of Cx-mGluR5 KO mice. Low-magnification images of double immunofluorescence with anti-mGluR5 and anti-CB1 antibodies on mPFC sections of control and Cx-mGluR5 KO mice (**A1**, **B1**). **A2** and **B2** depict merged staining of mGluR5, CB1 and DAPI within the white square from the left panel. The expression of mGluR5 was dramatically reduced in the cortex of Cx-mGlu5 KO mice (**B3**), while the CB1 expression was similar to littermate controls (A4, B4). (Roman numerals indicated different layers in mPFC. Scale bars in A1, B1∶500 µm; A2, A3, A4, B2, B3, B4∶50 µm.

mGluR5 and CB1R are present in the mouse medial prefrontal cortex (mPFC) and pharmacological studies suggest that CB1R-dependent long-term depression (LTD) in mPFC depends on mGluR5 function [65]. Here we explored the relative localization of mGluR5 and CB1R in mPFC by immunofluorescence staining, and then examined whether mGluR5/eCB-dependent LTD is still present in the Cx-mGluR5 KO mice. mGluR5 and CB1R double immuno-staining was conducted to examine mGluR5 and CB1R distribution in the mPFC of 2-month-old Cx-mGlu5 KO mice (n = 4) and their littermates (n = 4) (**Fig. 2**). mGluR5 immunoreactivity was evident in all cortical layers of control animals (**Fig. 2A** **1**), as previously reported [65]. In particular, prominent mGluR5-positive puncta were seen in cortical layers V/VI of the prelimbic area of mPFC (PrPFC) in control animals (**Fig. 2A** **2–3**). Minimal mGluR5 immunoreactivity was observed in the same area in Cx-mGlu5 KO mice (**Fig. 2B** **1–3**). CB1R immunoreactivity was present as dense meshwork of axons in layers II–III and in layer V–VI (**Fig. 2** **A4, B4**) as described [65]. CB1R expression patterns and abundance were similar between control and Cx-mGlu5 KO mice. These results demonstrate that deletion of mGluR5 from cortical glutamatergic neurons reduced mGluR5 levels but do not grossly alter CB1R expression or distribution.

**Figure 3:**
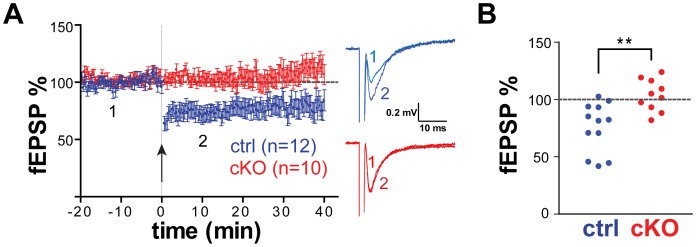
LTD formation is impaired in layer V of mPFC of Cx-mGlu5 KO mice. (**A**). Averaged fEPSP time courses of the LTD experiments in which the 10-minutes, 10 Hz protocol was applied to slices from control (blue; n = 12 at 6 mice) and cKO mice (red; n = 10 at 5 mice). Stimulation is indicated by the arrow. Right panel: sample traces are representative of averaged fEPSP’s (from six recordings) recorded before (1) and after (2) 10 Hz stimulation. (**B**). Scatter plots showed a significant change of %fEPSP between control and cKO mice (**p<0.01, Student's t-test). The average magnitude of LTD was assessed 20 minutes after stimulation. fEPSP amplitudes are expressed as percent of the baseline amplitude.

eCB-dependent LTD of the glutamatergic synapses between layer II/III and layer V/VI of PrPFC can be triggered by a 10 minute, 10 Hz stimulation of layer II/III afferents projecting onto layer V/VI [65]. eCB-LTD formation depends on CB1 and mGluR5, but not NMDA, D1, or D2 receptors. To examine whether this eCB-LTD formation is defective in Cx-mGlu5 PrPFC, field potential recordings were conducted with acute mPFC brain slices prepared from 4–6 week old Cx-mGlu5 KO mice (10 slices from 5 animals) and their littermate controls (12 slices from 6 animals). Field excitatory post-synaptic potentials (fEPSPs) were recorded in the prelimbic layer V/VI while stimulating in layer II/III in the presence of the GABA-A antagonist, GABAzine (10 µM). Robust LTD of glutamatergic inputs was triggered with the 10 minute 10 Hz stimulation paradigm in wild-type littermates (**Fig. 3A**). However, LTD formation could not be induced in Cx-mGlu5 KO mice. The magnitude of depression after the LTD stimulation paradigm was significantly different between control and Cx-mGlu5 KO mice (**Fig. 3B**; p = 0.002; fEPSP% after LTD stimuli: control, 75.5±6.2%; cKO: 103.2±4.4 of baseline responses). Thus, mGluR5-dependent eCB-LTD formation is impaired in the PrPFC of Cx-mGlu5 KO mice.

### Cx-mGlu5 KO Mice are Hyperactive in a Novel Environment

**Figure 4:**
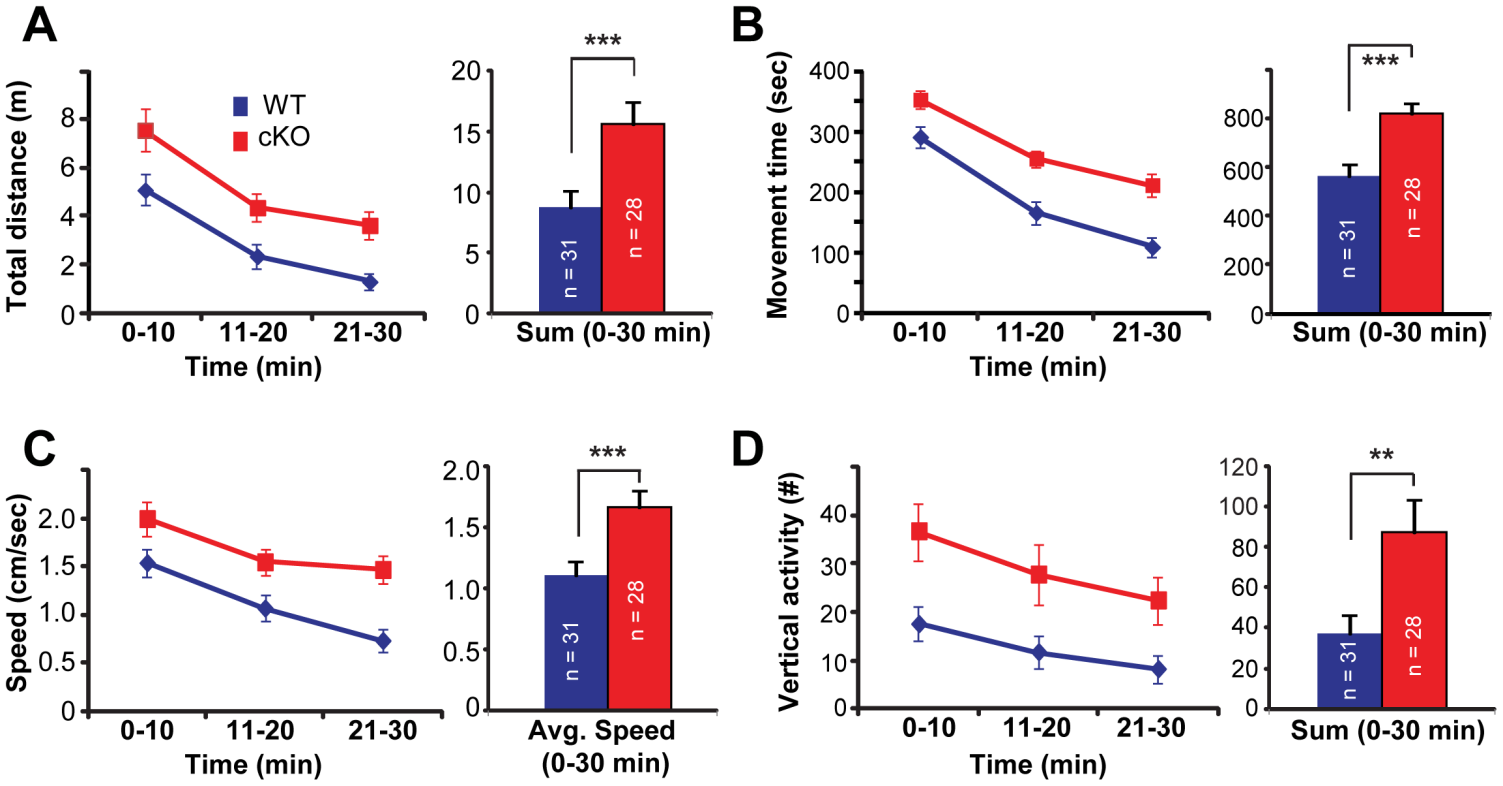
Cx-mGlu5 KO mice exhibit enhanced locomotor responses to a novel environment. (**A**) Total distance traveled, (**B**) movement time, (**C**) movement speed, and (**D**) vertical activity (numbers of rearing) during 30 minutes of open field assays are presented in 10-minutes bins as well as 0–30 minutes in bar graphs (blue for wild type and red for Cx-mGluR5 KO mice).

To examine whether ablation of mGluR5 from the glutamatergic neurons in the cortex leads to an increase in novelty induced locomotion, open field exploration tests [54] were conducted with Cx-mGlu5 KO and their littermate control mice, both of whom were naïve to the open field arena. The exploration reactivity to the novel environment and within-session habituation were analyzed by several measures in 3 time brackets using three-way ANOVA with repeated measures (Fig. 4). There was no genotype × gender interaction for total distance traveled (*F*_1, 55_ = 0.955, *p* = 0.333), movement time (*F*_1,55_ = 0.889, *p* = 0.350), average horizontal speed (*F*_1,55_ = 1.731, *p* = 0.194), or vertical activity (*F*_1,55_ = 0.043, *p* = 0.252). Thus, gender data were combined and analyzed with two-way ANOVA with repeated measures. Cx-mGlu5 KO mice are hyperactive (Fig. 4 and Table 1) with significant increases in total distance traveled (Fig. 4A; *F*_1,55_ = 9.001, *p* = 0.004), movement time (Fig. 4B; *F*_1,55_ = 17.230, *p*<0.001), average horizontal speed (Fig. 4C; *F*_1,55_ = 9.180, *p* = 0.004), and vertical activity (Fig. 4D; *F*_1,55_ = 7.292, *p = *0.009). Despite the hyperactivity, Cx-mGlu5 KO mice habituated to the novel environment with time. Both Cx-mGlu5 KO and control littermates decreased their exploratory activity as testing progressed as indicated by decreases in total distance traveled (*F*_1.46, 83.16_ = 74.248, *p*<0.001), movement time (*F*_1.51, 82.80_ = 129.86, *p*<0.001), average horizontal speed (*F*_1.65, 90.87_ = 34.196, *p*<0.001), and vertical activity (*F*_1.80, 99.20_ = 17.685, *p*<0.001).

**Table 1:** Behavior data summary for Cx-mGluR5 in 129/C57 background.

Behavioral paradigm	Measurement	Male		Female		Genotype effect	Gendereffect	Male+Female		Genotype effect
		WT	KO	WT	KO	p value	p value	WT	KO	p value
**Open Field**	total distance traveled (cm)	653.1±138.0 (15)	1566.0±182.3 (15)	1079.4±221.0 (16)	1543.7±356.2 (13)	0.004*	0.383	873.12±135.70 (31)	1555.61±188.19 (28)	0.004*
	movement time (s)	500.8±57.3 (15)	815.1±45.1 (15)	627.0±68.6 (16)	825.0±72.8 (13)	<0.001*	0.275	565.93±45.69 (31)	819.69±40.73 (28)	<0.001*
	movement speed (cm/s)	1.0±0.1 (15)	1.8±0.2 (15)	1.3±0.2 (16)	1.6±0.3 (13)	0.004*	0.721	1.11±0.12 (31)	1.67±0.14 (28)	0.004*
	vertical activity	24.5±8.1 (15)	94.9±21.1 (15)	49.4±15.2 (16)	77.5±26.0 (13)	0.009*	0.836	37.32±8.93 (31)	86.82±16.32 (28)	0.008
	center zone distance (cm)	53.7±18.6 (15)	118.9±42.1 (15)	102.9±44.8 (16)	225.6±100.1 (13)	0.097	0.165	79.11±24.80 (31)	168.16±51.63 (28)	0.114
	center zone duration (s)	28.4±6.4 (15)	39.0±10.2 (15)	34.4±9.6 (16)	66.1±21.7 (13)	0.096	0.190	31.53±5.77 (31)	51.61±11.51 (28)	0.114
	center distance ratio	0.06±0.02 (15)	0.07±0.02 (15)	0.06±0.01 (16)	0.10±0.03 (13)	0.269	0.538	0.06±0.01 (31)	0.08±0.02 (28)	0.285
**RotoRod**	latency to fall (s) trial 1	90.6±11.6 (11)	68.9±12.0 (10)	n.a.	n.a.	0.210	n.a.	n.a.	n.a.	n.a.
	latency to fall (s) trial 2	149.6±19.1 (11)	77.3±20.1 (10)	n.a.	n.a.	0.017*	n.a.	n.a.	n.a.	n.a.
	latency to fall (s) trial 3	155.2±22.9 (11)	128.2±26.9 (10)	n.a.	n.a.	0.451	n.a.	n.a.	n.a.	n.a.
	latency to fall (s) trial 4	163.5±23.1 (11)	135.8±25.6 (10)	n.a.	n.a.	0.432	n.a.	n.a.	n.a.	n.a.
	latency to fall (s) trial 5	197.4±26.9 (11)	187.4±24.3 (10)	n.a.	n.a.	0.788	n.a.	n.a.	n.a.	n.a.
	latency to fall (s) trial 6	220.4±22.2 (11)	201.2±30.4 (10)	n.a.	n.a.	0.612	n.a.	n.a.	n.a.	n.a.
	latency to fall (s) trial 7	230.8±26.4 (11)	204.3±30.4 (10)	n.a.	n.a.	0.516	n.a.	n.a.	n.a.	n.a.
	latency to fall (s) trial 8	234.3±23.5 (11)	227.6±27.6 (10)	n.a.	n.a.	0.855	n.a.	n.a.	n.a.	n.a.
	latency to fall (s) day 1	139.68±20.97 (11)	102.55±23.13 (10)	n.a.	n.a.	0.171	n.a.	n.a.	n.a.	n.a.
	latency to fall (s) day 2	220.70±24.33 (11)	205.13±27.58 (10)	n.a.	n.a.	0.234	n.a.	n.a.	n.a.	n.a.
**Prepulse Inhibition**	% PPI at 74 dB prepulse	29.9±6.1 (25)	21.8±6.2 (20)	16.3±4.2 (14)	16.0±6.9 (14)	0.285	0.135	25.02±4.26 (39)	19.42±4.56 (34)	0.373
	% PPI at 78 dB prepulse	51.0±6.4 (25)	42.2±6.7 (20)	30.4±5.1 (14)	28.1±6.7 (14)	0.513	0.012*	43.60±4.74 (39)	36.41±4.87 (34)	0.295
	% PPI at 82 dB prepulse	65.9±4.7 (25)	61.3±5.3 (20)	49.1±5.5 (14)	46.9±7.0 (14)	0.418	0.007*	59.88±3.78 (39)	55.36±4.34 (34)	0.433
	normalized startle response (A.U./g body weight)	664.2±103.6 (25)	934.4±135.9 (20)	700.6±91.5 (14)	686.6±98.4 (14)	0.549	0.377	677.29±73.34 (39)	832.36±90.96 (34)	0.184
**Homecage**	total activity in dark phase(# beam breaks)	3054.4±206.3 (14)	3395.6±279.2 (14)	n.a.	n.a.	0.335	n.a.	n.a.	n.a.	n.a.
	total activity in light phase(# beam breaks)	1196.4±109.1 (14)	1386.9±196.6 (14)	n.a.	n.a.	0.405	n.a.	n.a.	n.a.	n.a.

Values are listed as mean ± SEM (number of animals analyzed). Abbreviations: A.U., arbitrary unit;

* , p<0.05.

The ratio of center distance to total distance traveled and percentage of time spent in the center zone measured in the open field assay provide measures of anxiety-related responses to a bright and open arena [66], [67]. No genotype × gender interactions (Greenhouse-Geisser corrected) were found for the center zone distance traveled (*F*_1,55_ = 0.272, *p* = 0.604), center zone duration (*F*_1,55_ = 0.715, *p* = 0.401), or center distance ratio (*F*_1,55_ = 0.729, *p* = 0.397). No difference in the ratio of center distance to total distance traveled (center distance ratio: *F*_1,55_ = 1.248, *p* = 0.269) (Table 1) indicates that Cx-mGlu5 KO mice have a normal level of anxiety in the open field apparatus.

**Figure 5:**
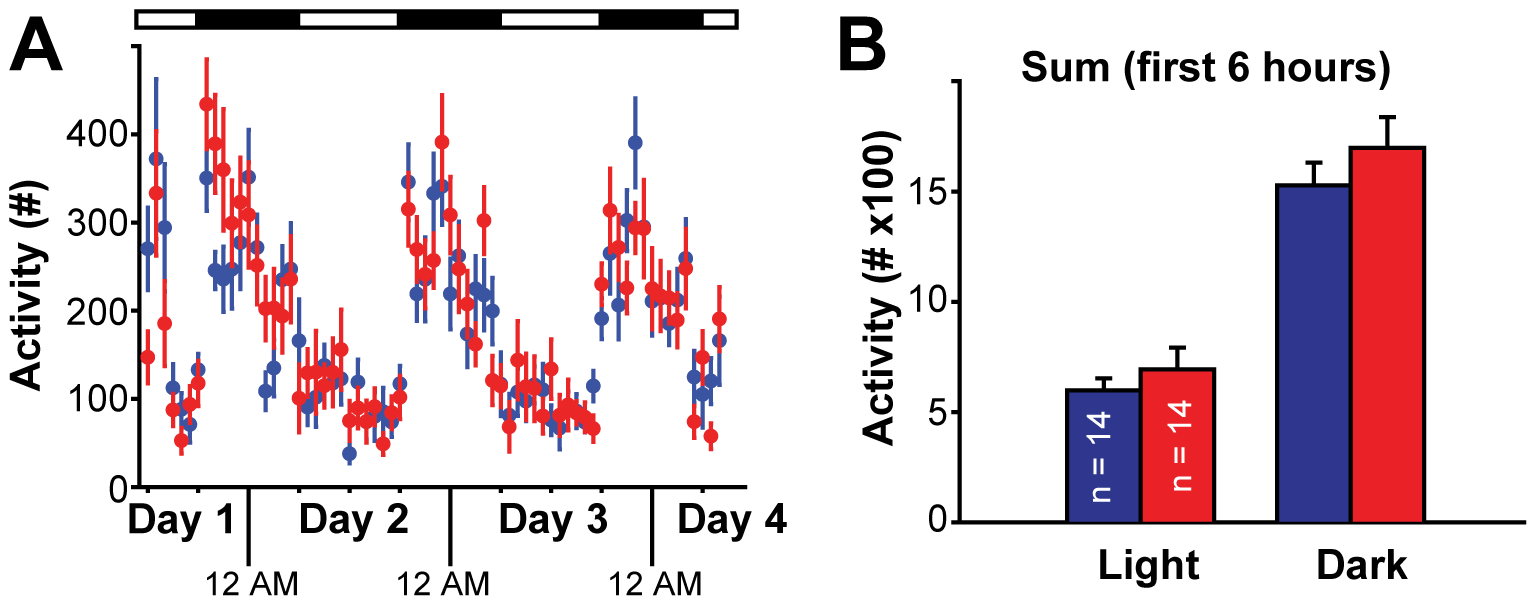
Cx-mGlu5 KO mice exhibit normal locomotion in familiar environment. (**A**) Home cage activity was plotted in one-hour bins. Light and dark phases over twenty-four hours are indicated as open and closed boxes, respectively. (**B**) Summary for the total activity during the first six hours in the light or dark phases.

To confirm that the observed hyperactivity in Cx-mGlu5 KO mice was due to novelty, we assessed their diurnal activity levels in the home cage environment for 72 hours. In this setting, mice were individually placed into standard polypropylene cages similar in size and containing the same bedding as their home cages. In contrast to the hyper-locomotion phenotype observed in the open field assay, Cx-mGlu5 KO mice showed normal activity levels in the home cage setting (Fig. 5, Table 1). Both control and Cx-mGlu5 KO mice were more active in the dark phase than light phase. The activity level during either dark (*t*_26_ = −0.98, *p* = 0.335) or light phase (*t*_26_ = −0.85, *p* = 0.405) was comparable between Cx-mGlu5 KO and littermate control mice. Taken together, these results suggest that Cx-mGlu5 KO mice are hyperactive relative to their wild-type littermates only in a novel environment.

**Figure 6:**
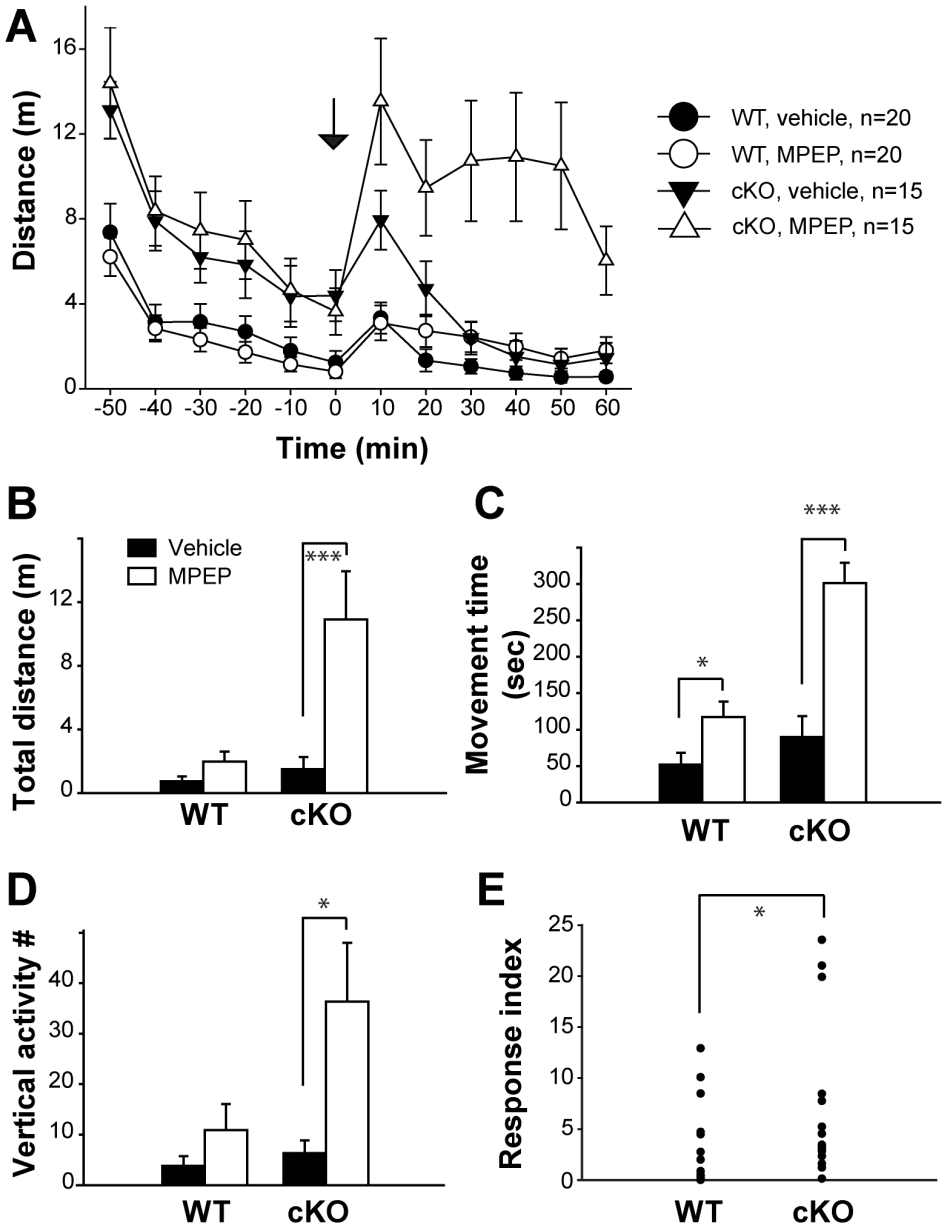
Enhanced activity levels in the open field assay for Cx-mGlu5 mice after MPEP administration. (**A**) Total distance traveled is presented in 10-minute time bins before and after vehicle or MPEP injection, ‘0′ represents injection time (arrow). (**B**) Total distance traveled (WT: vehicle = 0.74±0.31 m, MPEP = 1.98±0.63 m; KO: vehicle = 1.51±0.74 m, MPEP = 10.92±3.03 m), (**C**) movement time (WT: vehicle = 51.65±16.33 s, MPEP = 117.16±21.29 s; KO: vehicle = 89.58±28.76 s, MPEP = 301.53±27.97 s), and (**D**) vertical activity (WT: vehicle = 3.85±1.87, MPEP = 10.90±5.17; KO: vehicle = 6.33±2.56, MPEP = 36.33±11.69), are summarized as a 10-minute time bin, 31–40 minute after injection. (**E**) Summaries of response index for fold changes in distance travelled induced by MPEP (WT = 2.27±0.41, KO = 3.37±0.31). Data are presented as mean ± SEM.

Acute blockade of mGluR5 signaling using the noncompetitive mGluR5 antagonist 2-methyl-6-(phenylethynyl)-pyridine (MPEP) induces hyperactivity in wildtype mice when tested in an open-field arena [37]. To test whether mGluR5 signaling in cortical glutamatergic neurons mediates the hyper-kinetic effect of MPEP, we subjected Cx-mGlu5 KO and littermate control mice to 40 mg/kg MPEP or vehicle and examined their locomotion with the open field assay. Each mouse was placed in the open field arena and habituated to the chamber for 1 hour before drug injection, and activity levels were followed for another hour. Activity levels during the 31–40 minutes post-injection time bin were compared by two-way ANOVA (genotype × drug). To our surprise, Cx-mGlu5 KO mice had a greater increase in activity after MPEP, compared to their control littermates (Fig. 6). Statistical analysis showed that there were overall significant genotype and drug effects in total distance traveled (Fig. 6A,B; genotype: *F*_1,66_ = 8.477, *p* = 0.001; drug: *F*_1,66_ = 14.400, *p*<0.001). In addition, there was a significant genotype × drug effect (*F*_1,66_ = 8.477, *p* = 0.005), suggesting that the hyper-kinetic effects of MPEP are enhanced in Cx-mGlu5 KO mice. Response indexes were significantly higher in Cx-mGluR5 KO mice compared to control mice (Fig. 6E; *p*≤0.05). Similarly, there were overall significant genotype and drug effects in the time spent moving (Fig. 6C; genotype: *F*_1,66_ = 22.862, *p*<0.001, drug: *F*_1,66_ = 35.614, *p*<0.001), as well as a significant genotype × drug effect (*F*_1,66_ = 9.92, *p* = 0.002). Vertical activity showed a genotype effect (Fig. 6D; *F*_1,66_ = 5.302, *p* = 0.024) as well as a positive drug effect (*F*_1,66_ = 9.338, *p* = 0.003), but no genotype × drug interaction effect. Taken together, our observations suggest that mGluR5 signaling in cortical glutamatergic neurons does not mediate the hyper-kinetic effect of MPEP.

### Cx-mGlu5 KO Mice are Sensitive to Methylphenidate Treatment

**Figure 7:**
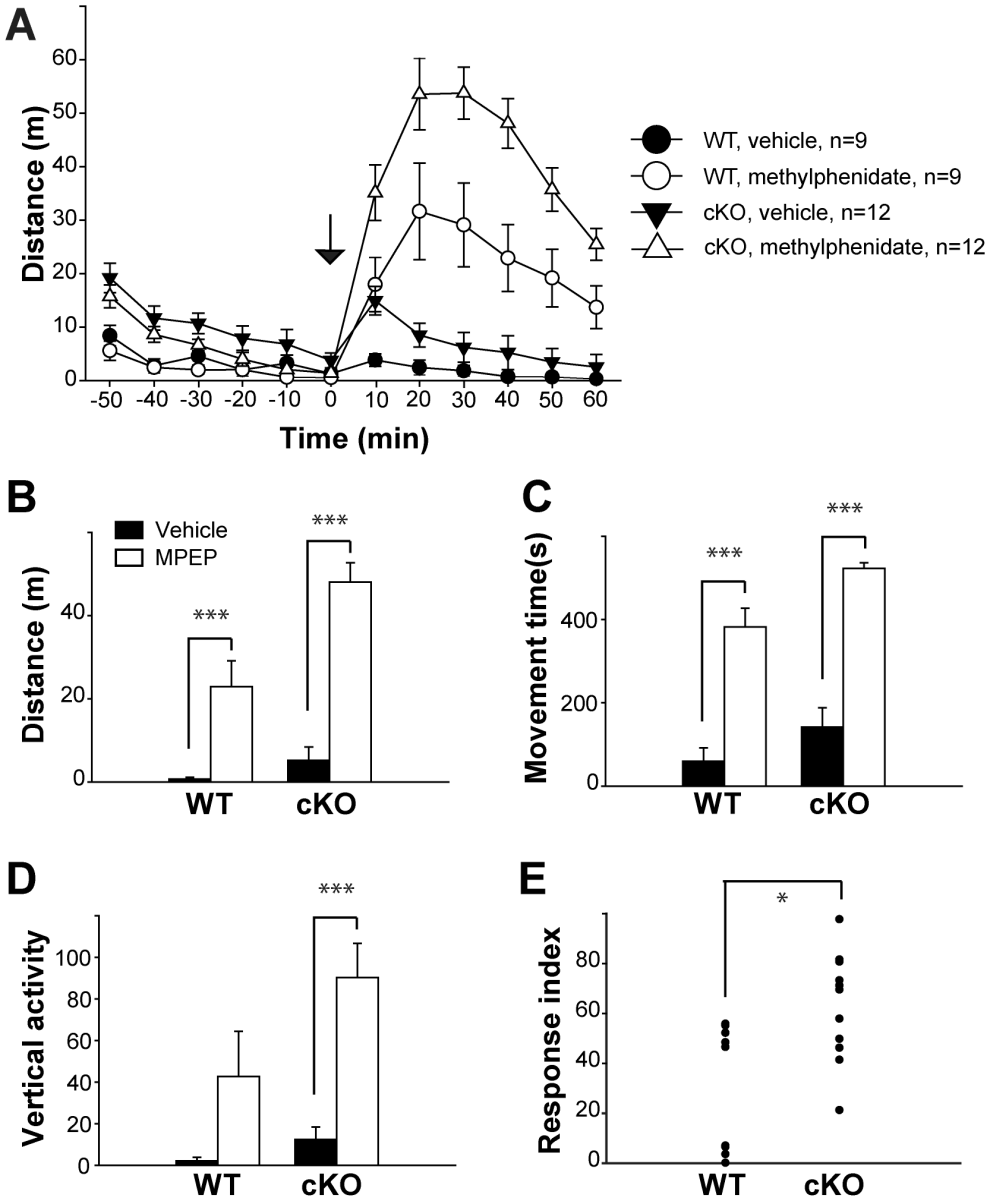
Enhanced activity levels in the open field assay for Cx-mGlu5 mice after methylphenidate administration. (**A**) Total distance traveled is presented in 10-minute time bins before and after vehicle or methylphenidate injection, ‘0′ represents injection time (arrow). (**B**) Total distance traveled (WT: vehicle = 0.74±0.42 m, methylphenidate = 22.91±6.27 m; KO: vehicle = 5.25±3.16 m, methylphenidate = 48.08±4.63 m), (**C**) movement time (WT: vehicle = 59.38±32.02 s, methylphenidate = 382.74±44.53 s; KO: vehicle = 141.89±46.33 s, methylphenidate = 522.93±13.73 s), and (**D**) vertical activity (WT: vehicle = 2.11±0.11, methylphenidate = 42.78±21.66; KO: vehicle = 12.42±6.03, methylphenidate = 90.33±16.36) are summarized as a 10-minute time bin, 31–40 minute after injection. (**E**) Summaries of response index for fold changes in distance travelled triggered by methylphenidate (WT = 30.67±8.39, KO = 64.36±6.20). Data are presented as mean ± SEM.

Psychostimulants characteristically enhance motor activity in normal individuals by increasing monoamine (e.g. dopamine, norepinephrine, and serotonin) neurotransmission [68]. Paradoxically, these compounds exert a calming effect in ADHD patients and are efficacious in various ADHD animal models. Methylphenidate, also known as Ritalin, is a commonly used psychostimulant to treat ADHD patients [69]. To assess methylphenidiate’s actions involve cortical mGluR5, we evaluated the effects of methylphenidate on locomotion using the open field assay. Cx-mGlu5 KO and their littermate control mice were habituated to the open field test chamber for 1 hour prior to receiving 8 mg/kg methylphenidate or vehicle. Methylphenidate strongly stimulated both Cx-mGlu5 KO mice and their littermate controls (**Fig. 7**). Significant increases in total distance traveled (**Fig. 7A**), movement time, stereotypic activity, and vertical activity were observed, with peak activity occurring 31–40 minutes after injection.

Activity levels during the 31–40 minute post-injection time bin were compared by two-way ANOVA (genotype × drug). Cx-mGlu5 KO mice had a greater response in activity after methylphenidate, compared to their control littermates. Statistical analysis showed that there were overall significant genotype and treatment effects in total distance traveled (**Fig. 7B**; genotype: *F*_1,40_ = 12.955, p = 0.001; drug: *F*_1,40_ = 62.141, *p*<0.001). Two-way ANOVA (genotype × drug) showed a significant increase in distance traveled for Cx-mGlu5 KO mice compared to control mice after drug injection (*F*_1,40_ = 6.284, p = 0.016; **Fig. 7E**). Significant genotype effects were found on time spent moving (**Fig. 7C**; *F*_1,40_ = 9.720, p = 0.003) and vertical activity (**Fig. 7D**; *F*_1,40_ = 4.646, p = 0.037). Neither movement time, nor vertical activity had a genotype × drug interaction effect. These observations suggest that methylphenidate exacerbates the hyper locomotion seen in Cx-mGlu5 KO mice.

### Normal Sensorimotor Gating, Motor Coordination/learning in Cx-mGlu5 KO Mice

**Figure 8:**
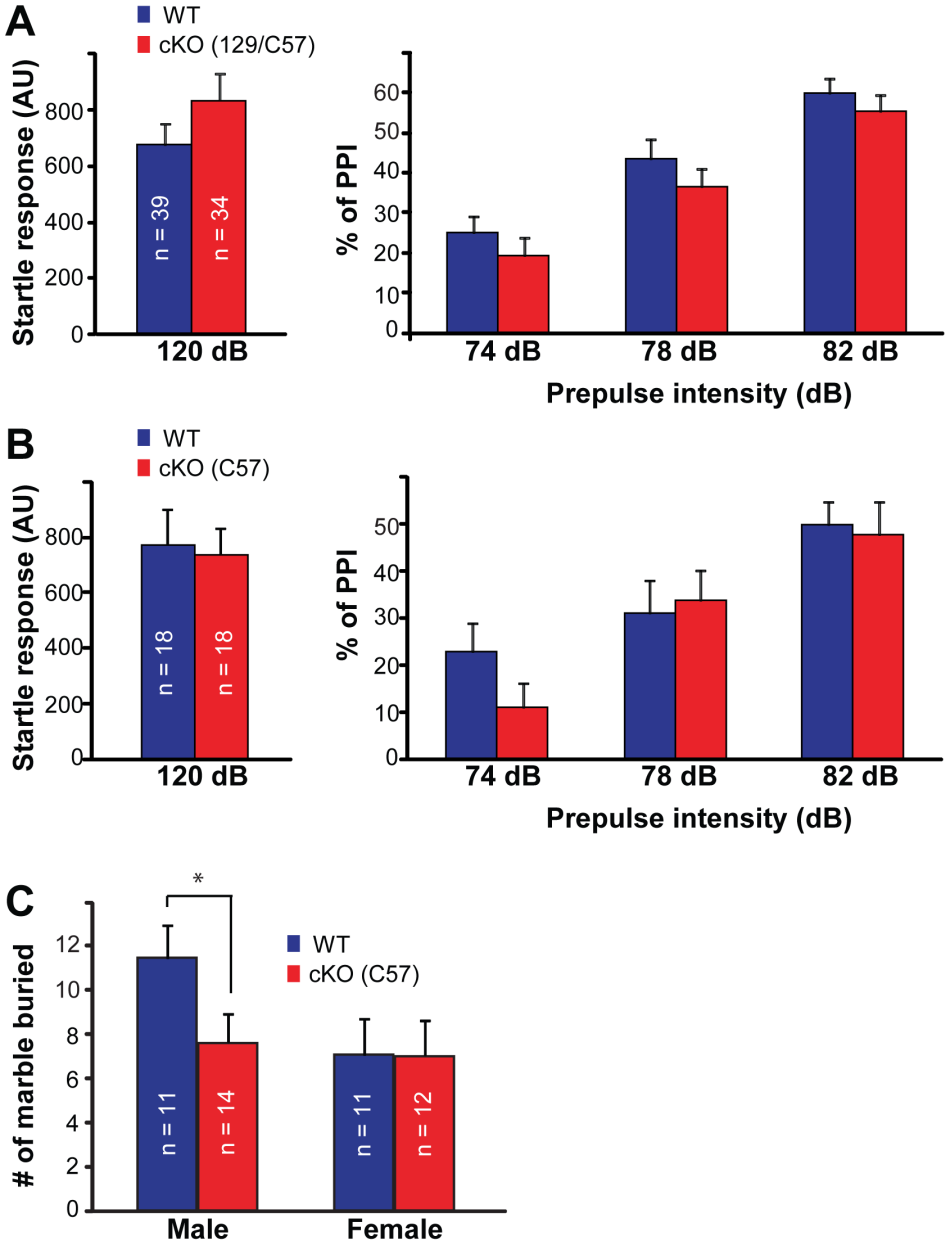
Cx-mGlu5 mice exhibit normal sensorimotor gating and a slight reduction in repetitive behaviors. (**A,B**) Sensorimotor gating was measured by prepulse inhibition (PPI) of the acoustic startle response in Cx-mGlu5 KO mice in C57/129 mixed (**A**) or in C57BL/6 background (**B**) and their littermate controls. Summaries of the maximum startle response to a 120 dB white noise sound burst are shown in the left panel. Summaries for the inhibition of the acoustic startle response by either one of three prepulse levels (74, 78 and 82 dB) are shown on the right. (**C**) Summaries for the number of marbles buried by Cx-mGlu5 KO and control mice in C57BL6 background.

The prepulse inhibition of acoustic startle reflex (PPI) provides an operational measure of sensory-motor gating processes in human and mice. To investigate the involvement of Cx-mGlu5 in sensorimotor gating, we performed the PPI test on male and female Cx-mGlu5 KO and their littermate control mice. All mice tested in the present study showed a startle response, and their data were thus included in the analysis. For startle response, there was no significant difference found in the response to maximum stimulus at 120 dB (genotype: *F*_1,69_ = 1.162, *p* = 0.285; gender: *F*_1,69_ = 0.792, *p* = 0.377; genotype × gender: *F*_1,69_ = 1.430, *p* = 0.236; gender data were pooled and shown in **Fig. 8A**). For PPI, there was a main effect of prepulse level (*F*_2,138_ = 188.01, *p*<0.001) as expected. As the prepulse level increases, there is generally a greater level of suppression generated in response to the startle stimulus. There was no genotype, gender, genotype × prepulse level, or gender × prepulse level interaction effects for the Cx-mGlu5 mice. These results demonstrate that mGluR5 expression in cortical glutamatergic neurons is not required for sensorimotor gating.

To explore the potential role of mGluR5 in cortical glutamatergic neurons in motor coordination/control and balance, we tested Cx-mGlu5 KO and littermate control male mice on the accelerating rotarod. The mice were subjected to 4 trials per day for two consecutive days, and the averaged results of latency to fall for each day were analyzed using two-way ANOVA with repeated measures (genotype × day). Mice of both genotypes improved significantly over the 2 days of training (**Table 1**; *F*_3.8,71.6_ = 27.38, *p*<0.001). There were no differences in latency to fall between the genotypes (*F*_1,19_ = 0.92, *p* = 0.351), nor was there a genotype × day interaction (*F*_3.8,71.6_ = 0.94, *p* = 0.441), indicating that Cx-mGlu5 KO mice have normal motor coordination, and showed no difference in motor learning on this task compared to wild type mice.

### Normal Anxiety-like Behaviors, Social Interactions, and Learning in Cx-mGlu5 KO Mice

Mice in a C57BL/6 background are more suitable to study anxiety-related behaviors [50]. Thus, we back-crossed Cx-mGlu5 mice into the C57BL/6 background (see Materials and Methods) for 6–8 generations. For the following assays, Cx-mGlu5 KO mice in a C57BL6 background were used. Similar to Cx-mGlu5 KO mice in 129-C57BL/6 mixed background, the mGluR5 conditional KO mice in C57BL/6 background exhibited hyperactivity in the novel environment (**Table 2**) and showed normal sensorimotor gating in the PPI test (**Fig. 8B**). In addition to open field test, Cx-mGlu5 KO mice in the C57BL/6 background and their littermates were also subjected to elevated plus maze and light-dark exploration tests. In the elevated plus maze assay, we found that there was no overall difference between the genotypes or genders in the number of entries to the open arms (**Table 2**; genotype: *F*_1,23_ = 0.351, p = 0.559; gender *F*_1,23_ = 1.993, p = 0.171) or in the amount of time spent in the open arms (genotype: *F*_1,23_ = 0.033, p = 0.857; gender: *F*_1,23_ = 3.268, p = 0.084). In the light-dark exploration test both Cx-mGluR5 KO and littermate WT groups exhibited frequent transitions between the two compartments as expected for pure C57BL/6 mice (**Table 2**). There was no effect on genotype for transitions made between the light and dark environment (*F*_1,41_ = 0.311, p = 0.580) or for the percentage of time spent in the light chamber (*F*_1,41_ = 2.171, p = 0.148). In addition, no difference was found in circulating corticosteroid levels at rest between Cx-mGluR5 KO (53.1±4.2 ng/ml, n = 12) and control mice (46.3±3.7 ng/ml; n = 13). Thus, the basal activity in the Hypothalamic-Pituitary-Adrenal axis in Cx-mGluR5 mice is normal.

**Table 2:** Behavior data summary for Cx-mGluR5 in C57 background.

Behavioral paradigm	Measurement	Male		Female		Genotype effect	Gendereffect	Male+Female		Genotype effect
		WT	KO	WT	KO	p value	p value	WT	KO	p value
**Open Field**	total distance traveled (cm)	2716.2±269.3 (16)	5197.2±423.2 (17)	4326.0±589.4 (6)	5000.1±463.6 (7)	0.002*	0.139	3155.25±291.05 (22)	5139.75±323.95 (24)	<0.001*
	movement time (s)	342.0±21.4 (16)	379.9±42.1 (17)	395.2±60.7 (6)	467.5±49.6 (7)	0.233	0.120	356.54±22.43 (22)	405.29±33.57 (24)	0.212
	movement speed (cm/s)	8.68±0.93 (16)	18.84±4.32 (17)	11.3±0.3 (6)	11.0±0.7 (7)	0.190	0.486	9.40±0.72 (22)	16.55±3.13 (24)	0.038*
	vertical activity	186.6±29.8 (16)	223.5±23.9 (17)	212.2±57.4 (6)	200.6±52.1 (7)	0.745	0.001*	193.57±26.10 (22)	216.79±22.19 (24)	0.499
	center zone distance (cm)	623.4±105.4 (16)	1069.5±154.1 (17)	941.2±414.4 (6)	1156.9±240.7 (7)	0.193	0.973	710.10±133.68 (22)	1094.94±127.13 (24)	0.043*
	center zone duration (s)	227.8±37.5 (16)	296.6±36.4 (17)	235.2±129.3 (6)	327.0±75.5 (7)	0.117	0.333	229.83±42.64 (22)	305.48±33.05 (24)	0.164
	center distance ratio	0.20±0.03 (16)	0.21±0.03 (17)	0.17±0.06 (6)	0.24±0.04 (7)	0.315	0.758	0.19±0.02 (22)	0.22±0.02 (24)	0.417
**Elevated Plus Maze**	time spent in open arm (s)	146.7±29.0 (10)	160.8±20.5 (9)	211.1±34.6 (4)	208.3±17.1 (4)	0.857	0.084	165.07±23.68 (14)	175.40±16.01 (13)	0.654
	number of open arm entries	16.0±1.6 (10)	20.0±2.5 (9)	16.3±1.3 (4)	25.3±3.1 (4)	0.016*	0.281	16.07±1.17 (14)	21.62±2.00 (13)	0.275
	number of head dips	24.9±3.7 (10)	20.7±4.6 (9)	12.8±5.6 (4)	26.8±3.8 (4)	0.346	0.556	21.43±3.35 (14)	22.54±3.41 (13)	0.854
	number of rearings	47.3±5.6 (10)	37.6±5.1 (9)	23.5±6.1 (4)	53.5±11.2 (4)	0.170	0.588	40.5±5.2 (14)	42.5±5.1 (13)	0.790
	number of groomings	8.5±1.3 (10)	6.4±1.4 (9)	9.5±1.7 (4)	10.5±3.2 (4)	0.781	0.191	8.79±1.04 (14)	7.69±1.40 (13)	0.568
	distance traveled in close arm (m)	8.3±0.6 (10)	6.8±0.6 (9)	7.4±0.7 (4)	9.7±0.5 (4)	0.554	0.167	8.0±0.5 (14)	7.7±0.6 (13)	0.680
**3 Chamber**	preference ratio - object (habituation)	1.39±0.15 (21)	1.16±0.19 (20)	n.a.	n.a.	0.341	n.a.	n.a.	n.a.	n.a.
	preference ratio - chamber (habituation)	1.19±0.15 (21)	1.11±0.12 (20)	n.a.	n.a.	0.678	n.a.	n.a.	n.a.	n.a.
	preference ratio - object (social)	2.57±0.39 (21)	2.82±0.45 (20)	n.a.	n.a.	0.677	n.a.	n.a.	n.a.	n.a.
	preference ratio - chamber (social)	1.98±0.36 (21)	1.47±0.21 (20)	n.a.	n.a.	0.240	n.a.	n.a.	n.a.	n.a.
**Conditioned Fear**	context (% time freezing)	10.9±2.5 (9)	21.1±6.9 (9)	16.0±3.9 (8)	16.9±6.6 (8)	0.152	0.933	13.32±2.26 (17)	19.11±4.68 (17)	0.274
	CS – uncued (% time freezing)	2.90±1.10 (9)	12.17±4.63 (9)	3.00±1.01 (8)	2.21±0.74 (8)	0.114	0.067	2.94±0.73 (17)	7.48±2.71 (17)	0.116
	CS – cued (% time freezing)	66.93±3.10 (9)	70.23±3.27 (9)	65.52±6.82 (8)	55.83±5.52 (8)	0.508	0.114	66.26±3.49 (17)	63.45±3.51 (17)	0.574
**Light-Dark**	dark-duration (s)	478.70±25.04 (10)	455.24±30.89 (14)	457.56±21.63 (10)	400.21±28.31 (11)	0.160	0.184	468.13±16.29 (20)	431.03±21.62 (25)	0.196
	dark-entry count	25.20±5.33 (10)	25.64±4.37 (14)	39.0±6.6 (10)	46.7±6.4 (11)	0.476	0.004*	32.10±4.43 (20)	34.92±4.24 (25)	0.651
	dark-latency (s)	68.6±36.9 (10)	18.4±10.7 (14)	39.5±17.8 (10)	34.1±17.0 (11)	0.197	0.297	54.05±20.23 (20)	25.28±9.52 (25)	0.177
	light-duration (s)	118.3±23.7 (10)	143.6±31.0 (14)	142.3±21.6 (10)	199.6±28.3 (11)	0.148	0.571	130.33±15.85 (20)	168.26±21.68 (25)	0.184
	light-entry count	29.9±6.4 (10)	26.8±4.4 (14)	39.2±6.6 (10)	46.8±6.4 (11)	0.703	0.366	34.55±4.58 (20)	35.60±4.19 (25)	0.867
**Footslip Assay**	number of footfalls	307.9±29.3 (10)	372.3±29.9 (8)	n.a.	n.a.	0.148	n.a.	n.a.	n.a.	n.a.
	footfalls: time active (s)	70.1±9.9 (10)	80.5±13.7 (8)	n.a.	n.a.	0.535	n.a.	n.a.	n.a.	n.a.
	footfalls: mean active duration (s)	0.2±0.0 (10)	0.2±0.1 (8)	n.a.	n.a.	0.687	n.a.	n.a.	n.a.	n.a.
	footfalls: activation frequency	0.5±0.1 (10)	0.6±0.1 (8)	n.a.	n.a.	0.149	n.a.	n.a.	n.a.	n.a.
	distance traveled (m)	10.4±1.0 (10)	15.4±2.1 (8)	n.a.	n.a.	0.031*	n.a.	n.a.	n.a.	n.a.
**Prepulse Inhibition**	% PPI at 74 dB prepulse	30.7±5.0 (8)	18.8±5.8 (8)	20.5±10.3 (9)	9.3±7.4 (9)	0.142	0.208	25.30±5.91 (17)	13.78±4.77 (17)	0.139
	% PPI at 78 dB prepulse	35.8±9.1 (8)	41.4±10.1 (8)	31.4±10.7 (9)	26.6±9.1 (9)	0.967	0.330	33.45±6.89 (17)	33.55±6.81 (17)	0.991
	% PPI at 82 dB prepulse	48.6±6.9 (8)	50.4±10.0 (8)	52.9±7.6 (9)	45.4±11.3 (9)	0.758	0.967	50.86±5.05 (17)	47.73±7.38 (17)	0.729
	normalized startle response (A.U./g body weight)	1099.9±228.8 (8)	846.1±131.0 (8)	482.9±76.5 (9)	598.5±121.5 (9)	0.638	0.006*	773.23±135.09 (17)	715.02±91.64 (17)	0.724
**Marble Burying**	marbles buried	11.5±1.4 (11)	7.6±1.0 (14)	7.1±1.1 (11)	7.0±1.3 (12)	0.109	0.041*	9.27±0.99 (22)	7.35±0.778 (26)	0.129

Values are listed as mean ± SEM (animals number). Abbreviations: A.U., arbitrary unit;

* , p<0.05.

To examine social interactions in Cx-mGlu5 KO mice, a modified automated three-chambered social approach task [70] was conducted. Distance traveled, time spent traveling, and transitions between chambers were comparable between wildtype and Cx-mGlu5 KO mice during both the habituation phase and the social phase. No differences in the chamber preference ratios (**Table 2**; *t*_39_ = 1.19, p = 0.24) or the sniffing preference ratios (*t*_39_ = 0.42, p = 0.67) were found. Next, we examined Pavlovian learning in Cx-mGlu5 KO mice using the conditioned fear paradigm associating contextual or auditory cues with mild foot shock. Cx-mGlu5 KO mice exhibited normal levels of freezing for both contextual and auditory cue-based fear tests performed 24 hours after training (**Table 2**). No significant genotype, gender or interaction effects were found in the context test (genotype: *F*_1,30_ = 1.077, p = 0.308; gender: *F*_1,30_ = 1.077, p = 0.308; genotype × gender: *F*_1,30_ = 0.773, p = 0.386) or in the freezing behavior for auditory cues (genotype: *F*_1,30_ = 2.156, p = 0.152; gender: *F*_1,30_ = 0.347, p = 0.560; genotype × gender: *F*_1,30_ = 0.084, p = 0.774). Thus, mGluR5 in cortical glutamatergic neurons is not required for social interactions or learning an aversive memory task.

### Repetitive Behavior is Reduced in Male Cx-mGlu5 KO Mice

Marble-burying behavior in mice is an indicative measure of highly repetitive digging and this repetitive behavior persists with little change across multiple exposures [63]. This behavior is genetically regulated, not correlated with other anxiety-like traits and not stimulated by novelty [63]. We found that there is significant gender effect on number of marbles buried (**Table 2**, genotype: *F*_1,44_ = 2.680, p = 0.109; gender: *F*_1,44_ = 4.411, p = 0.041; genotype × gender: *F*_1,44_ = 2.436, p = 0.126). Analyzing the genders separately using student’s t-test to compare Cx-mGluR5 KO and WT littermate mice, we found that Cx-mGlu5 KO male mice buried fewer marbles than their male littermates (**Fig. 8C**; p = 0.03), but there was no difference in female mice between the two genotypes. In wildtype mice, 40 mg/kg MPEP treatment reduces repetitive digging behavior by more than 80% [53]. We observed a small but significant reduction in marble burying behaviors in male but not female Cx-mGlu5 KO mice. These findings suggest that mGluR5 expression on cells other than cortical glutamatergic neurons has more influence on repetitive behaviors.

## Discussion

mGluR5 is expressed widely at high levels in the brain and abnormal mGluR5 signaling has been implicated in various neurological disorders. Here we found that deleting mGluR5 expression solely from cortical glutamatergic neurons leads to defective synaptic plasticity in the prefrontal cortex and results in an increase in novelty-induced locomotion in both 129-C57BL/6 mixed and C57BL/6 genetic backgrounds. Cx-mGluR5 KO mice show an exaggerated locomotor response to psychostimulants. Despite the significant increase in locomotion, Cx-mGluR5 KO mice still habituated to the novel environment. When Cx-mGluR5 KO mice were in a familiar environment, their activity levels during both the light and dark phases were similar to control mice. Cx-mGluR5 KO mice exhibited normal sensorimotor gating, motor coordination/learning, anxiety-like behaviors, social interactions, and fear-conditioning. Thus, the exaggerated hyper-locomotive responses of Cx-mGluR5 KO mice to a psychostimulant or novel environment is unlikely to be due to abnormal anxiety behaviors. Together these results suggest that mGluR5 signaling in cortical glutamatergic neurons is an essential part of the network responsible for regulating novelty-induced locomotion. In contrast, deleting mGluR5 expression in the cortical glutamatergic neurons has no detectable impact on sensorimotor gating, anxiety behaviors, social interactions, motor coordination/learning and fear conditioning behaviors.

### eCB-dependent LTD in PFC Requires mGluR5 Expression in Glutamatergic Neurons

One consequence of mGluR5 activation is an increase in the synthesis and mobilization of eCB’s and induction of CB1R-dependent synaptic plasticity [7], [8], [9]. A role for mGluR5 in eCB-dependent LTD in mPFC has been demonstrated using a pharmacological approach [65]. The defective eCB-dependent synaptic plasticity in Cx-mGluR5 KO PFC demonstrates the requirement of mGluR5 in pyramidal neurons for triggering this form of CB1R-dependent plasticity. CB1R in glutamatergic axonal terminals has been suggested to provide neuroprotection by preventing excessive excitatory neuronal activity by reducing glutamate release [71]. Removing CB1R from cortical glutamatergic neurons leads to a reduced seizure threshold in the kainic acid induced seizure model [71]. In preliminary studies we have also found a reduced seizure threshold in Cx-mGluR5 KO mice. Thus, defective CB1R signaling in Cx-mGluR5 KO mice is likely to lessen the inhibitory feedback control of glutamate, contributing to abnormal network excitability. It is possible that this defective synaptic plasticity or reduced inhibitory tone may contribute to the hyperactivity observed in Cx-mGluR5 KO mice.

### Cortical Glutamatergic mGluR5 Signaling Modulates Novelty-induced Locomotion

ADHD is a common neuropsychiatric disorder with onset at preschool age. 8–12 percent of school-aged children and around 4% adults have been diagnosed with ADHD [72], [73], [74], [75]. It has been hypothesized that problems in the circuits connecting the basal ganglia with prefrontal cortex, the caudate nucleus, and the globus pallidus are the primary causes underlying ADHD symptoms [76], [77]. Deletion of the mGluR5 gene has been found in a subset of ADHD patients [19], [20]. Here we found that removing mGluR5 expression from cortical glutamatergic neurons leads to an enhancement of novelty-triggered locomotion, a characteristic often found in ADHD animal models. Taken together, we propose that glutamatergic signaling through mGluR5 in the frontal cortex regulates the fronto-striatal circuitry to modulate novelty-induced locomotion.

Hyperactivity in ADHD animal models is typically normalized by treatment with psychostimulants like methylphenidate (known as Ritalin) (e.g. [78], [79]). Therapeutic doses of oral methylphenidate significantly increase extracellular dopamine in the human brain [80]. Unlike typical ADHD animal models, the locomotor hyperactivity in Cx-mGluR5 KO mice was not reduced by treatment with methylphenidate. Instead, Cx-mGluR5 KO mice exhibit significantly increased locomotion upon methylphenidate treatment.

Acute blockade of mGluR5 signaling through systemic MPEP treatment leads to a modest [53] or no increase [30] in locomotor activity. To our surprise, Cx-mGluR5 KO mice exhibit enhanced locomotion following MPEP treatment. One explanation for this finding is that mGluR5 on cells other than cortical glutamatergic neurons also participate in circuits regulating locomotion. The abundant mGluR5 expression in the striatum of Cx-mGluR5 KO mice makes striatal mGluR5 the most plausible target of MPEP in the Cx-mGluR5 KO mice. However, this reasoning alone doesn’t explain why MPEP has stronger effects on Cx-mGluR5 mice than wild type mice. It is plausible that deleting mGluR5 in cortical neurons in the Cx-mGluR5 KO mice prevents the proper establishment of inhibitory circuits to regulate mGluR5-containing striatal outputs.

The increased locomotor activity in Cx-mGluR5 KO mice in the open-field test environment was observed during the first visit for baseline studies as well as in the subsequent visits for MPEP or methylphenidate experiments. Thus, Cx-mGluR5 KO mice are more sensitive to test environment while home cage locomotor behavior remains unaffected. The hyperkinetic phenotype in Cx-mGluR5 KO mice is exaggerated to a greater degree than in wild type mice by acute systemic mGluR5 blockade and by the psychostimulant methylphenidate. The increased sensitivity to amphetamine is one characteristic of mania or mood disorders [81], [82]. Similar increases in novelty-induced locomotion and enhanced sensitivity to psychostimulants have been found in *Highper* mutant mice [83], an ENU-induced mutant line, and Shank3 transgenic mice with Shank3 overexpression (unpublished data).

Taken together, our findings demonstrate that mGluR5 signaling in cortical glutamatergic neurons is required to regulate novelty-induced locomotion but is not required for other behavioral parameters such as sensorimotor gating, anxiety, motor coordination/learning, social interactions or fear conditioning behaviors.
